# The Effects of *Turnip Mosaic Virus* Infections on the Deposition of Secondary Cell Walls and Developmental Defects in Arabidopsis Plants Are Virus-Strain Specific

**DOI:** 10.3389/fpls.2021.741050

**Published:** 2021-10-08

**Authors:** Silvia López-González, Concepción Gómez-Mena, Flora Sánchez, Mathias Schuetz, A. Lacey Samuels, Fernando Ponz

**Affiliations:** ^1^Centro de Biotecnología y Genómica de Plantas, Universidad Politécnica de Madrid-Centro Nacional Instituto Nacional de Investigación y Tecnología Agraria y Alimentaria, CSIC, Madrid, Spain; ^2^Instituto de Biología Molecular y Celular de Plantas, Universitat Politècnica de València-Consejo Superior de Investigaciones Científicas, Valencia, Spain; ^3^Department of Botany, University of British Columbia, Vancouver, BC, Canada

**Keywords:** turnip mosaic virus, secondary cell wall, developmental alterations, viral strains, viral chimeras

## Abstract

Two isolates of Turnip mosaic virus (UK 1 and JPN 1), representative of two different viral strains, induced differential alterations on secondary cell wall (SCW) development in *Arabidopsis thaliana*, suggesting cell-type specific effects of these viral infections. These potential effects were analyzed in inflorescence stems and flowers of infected plants, together with other possible cellular effects of the infections. Results obtained from macroscopic and histochemical analyses showed that infection with either virus significantly narrowed stem area, but defects in SCW were only found in JPN 1 infections. In flowers, reduced endothecium lignification was also found for JPN 1, while UK 1 infections induced severe floral cell and organ development alterations. A transcriptomic analysis focused on genes controlling and regulating SCW formation also showed notable differences between both viral isolates. UK 1 infections induced a general transcriptional decrease of most regulatory genes, whereas a more complex pattern of alterations was found in JPN 1 infections. The role of the previously identified viral determinant of most developmental alterations, the P3 protein, was also studied through the use of viral chimeras. No SCW alterations or creeping habit growth were found in infections by the chimeras, indicating that if the P3 viral protein is involved in the determination of these symptoms, it is not the only determinant. Finally, considerations as to the possibility of a taxonomical reappraisal of these TuMV viral strains are provided.

## Introduction

Viruses often induce disease symptoms in the infected host, which in plants include mottles or mosaics, yellowing, or stunted growth. These general symptoms have long been recognized, are easily observed and well documented. The underlying molecular and cellular mechanisms are not fully understood, although they are the subject of a sustained research, reviewed in detail a few years ago (Culver and Padmanabhan, 2007; Pallás and García, 2011). However, less information is available on those disease symptoms affecting specific developmental traits. Plant development is mostly post-embryonic, consisting of phases with marked transitions (Huijser and Schmid, 2011), consequently virus infections of plants have increased chances to affect traits that are characteristic of the developmental phase, as compared with organisms with an embryonic development.

In previous work, we have established a system that allows focusing on some of these developmental traits. Infections of *Arabidopsis thaliana* with *Turnip mosaic virus* (TuMV, a potyvirus) alter a number of developmental stages that we identified in practically all plant organs in a comparative analysis between virus strains (Sánchez et al., 2015). Notable differential alterations affected the elongation and erection of the main inflorescence stem, and inflorescence branching pattern. Thus, plants infected with the viral isolate UK 1 were not able to elongate a stem, or produced only a very short stem with no flowers, or a few sterile ones. Those infected with isolate JPN 1 elongated a creeping inflorescence stem, which is unable to maintain upright growth. The derived inflorescence was highly branched, giving the plants a bushy global appearance with the intertwined branches forming a net just over the rosettes. The viral protein P3 is associated with the elongation trait (López-González et al., 2020). No information is available about the viral determinant of the creeping and branching.

The developmental alterations described above are strongly suggestive of an impact of the TuMV infections on the normal formation of the cell walls of the plant main vertical axis cells. Cell walls are critical players in mediating upwards plant growth, physical structure, and global morphogenesis, in highly regulated processes (Houston et al., 2016). Both primary and secondary cell walls (SCWs) play roles in growth and physical structure, although those plant cells developing SCWs are mainly responsible for the latter. The cell and molecular biology of SCW deposition and regulation have been recently reviewed (Meents et al., 2018; Zhong et al., 2019). Since the infection by TuMV has a strong impact on Arabidopsis global morphogenesis, in this work, we have focused on the effects of the virus on SCW formation and composition, and in the transcriptomics of the genes involved in the regulation of their formation. This work deciphers the relationship between virus infections and their impact on plant SCWs.

## Materials and Methods

### Plant Growth Conditions and Virus Inoculations

*Arabidopsis thaliana* Col-0 plants were grown under controlled conditions: 21°C (day)/18°C (night) in 16-and 8-hcycles. Plants were inoculated at stage 1.08 (Boyes et al., 2001) with crude sap from virus-infected plants with TuMV UK 1 or TuMV JPN 1. In certain experiments, plants were inoculated with viral chimeras which interchange genomic fragments of the two strains. The description of the constructions of these chimeras and the inoculations to Arabidopsis plants have been previously published (Sánchez et al., 2015).

### Histological Analyses

Flower stalks from Arabidopsis (20days post inoculation) were cut 1cm above the base and embedded in 7% (w/v) low melting point agarose following the protocol described (Pradhan Mitra and Loqué, 2014). Agarose blocks were sectioned using a vibratome (Leica VT1200 S). Cross sections were stained with toluidine blue [0.05% (w/v)] or HCl-phloroglucinol staining solution [10% phloroglucinol in absolute ethanol (w/v) with the addition of concentrated HCl before use], or freshly mounted and imaged for lignin autofluorescence. Sections were treated with 0.01% calcofluor white M2r (Sigma) for cellulose visualization. Slides were observed with an optical microscope (Nikon Eclipse E600).

Flower buds were submerged in FAE solution [4% formaldehyde (v/v), 5% acetic acid (v/v), and 50% ethanol(v/v)], placed under vacuum for 10min and incubated overnight at 4°C. Samples were dehydrated in alcohol and embedded in acrylic resin (Technovit 7,100; Kulzer). For histological analysis, 1-μm transversal sections of the floral buds were obtained and stained with 0.05% toluidine blue in 0.1M phosphate buffer at pH 6.8 (O'Brien et al., 1964). Slides were observed with an optical microscope (Nikon Eclipse E600).

### Immunohistochemistry

For xylan immunostaining, flower stalks from mature Arabidopsis were cut 1cm above the base and fixed with FAE for 1h and washed with TBST solution [10mM Tris–HCl (pH 7.0), 0.25M NaCl, and 0.1% (w/v) Tween 20]. The sections were blocked with 0.2M PBS containing 5% (w/v) bovine serum albumin for 1h at room temperature, followed by incubation with anti-xylan LM10 rat monoclonal antibody (Plant Probes; (McCartney et al., 2005)) at 1:50 dilution overnight at 4°C with gentle shaking. Tissue samples were washed three times with TBST and incubated with anti-rat antibody conjugated to Alexa 488 (Invitrogen) at 1:100 dilution at room temperature for 1h. Imaging of stalk longitudinal sections was performed on Laser Scanning Microscope Leica TCS SP8 using conventional GFP settings (488/509).

### Scanning Electron Microscopy

Fresh samples were deep-frozen in slush nitrogen and attached to the specimen holder of a CryoTrans 1,500 Cryo-Preparation System (Oxford 127 Instruments, UK) interfaced with a JEOL JSM-5410 scanning electron microscope. Samples were gold-coated and observed at an accelerating voltage of 15keV.

### Gene Expression Analysis

Total RNA from stem longitudinal sections were extracted using the RNAeasy mini kit (Qiagen) following the manufacturer’s instructions. Buffer-inoculated plants were used as a control. For the analysis, the first internode starting from the rosettes was taken, the same part of the plant used for the histological analyses. To avoid possible cross-contaminations, a clean mortar and pestle was used for each treatment. cDNA was synthesized using the kit High Capacity RNA-to-cDNA (Life Technologies), according to the manufacturer’s instructions. Primers for RT-qPCR were designed using Primer3Plus (Supplementary Table S1). RT-qPCR was performed in a LightCycler 480 System (Roche), using LightCycler 480 SYBR green I master (Roche), following manufacturer’s instructions. Cycling conditions for amplifications were as follows. Initial activation step of 95°C for 5min followed by 45cycles of 95°C for 10s, 60°C for 10s, and 72°C for 15s. Changes in gene expression were determined using the 2^−ΔΔCT^ method (Livak and Schmittgen, 2001). Data were relativized to 1, thus errors in the controls were calculated using the Standard Deviation/Mock CT Average. Results were expressed as fold change relative to the housekeeping gene *Actin 8* (*AT1G49240*). The experiments were carried out in triplicate for each data point. In detail: Three-four plants (stems) were used to prepare one biological replica. In total, 10–12 plants per treatment were used, and there were three biological replicas. For each biological replica, three technical replicates were loaded in the plate. This experiment was done twice, with different biological material, and the results were similar.

## Results

### TuMV Infections Affect Arabidopsis Secondary Growth, Generating Narrow Stalks

**Figure 1:**
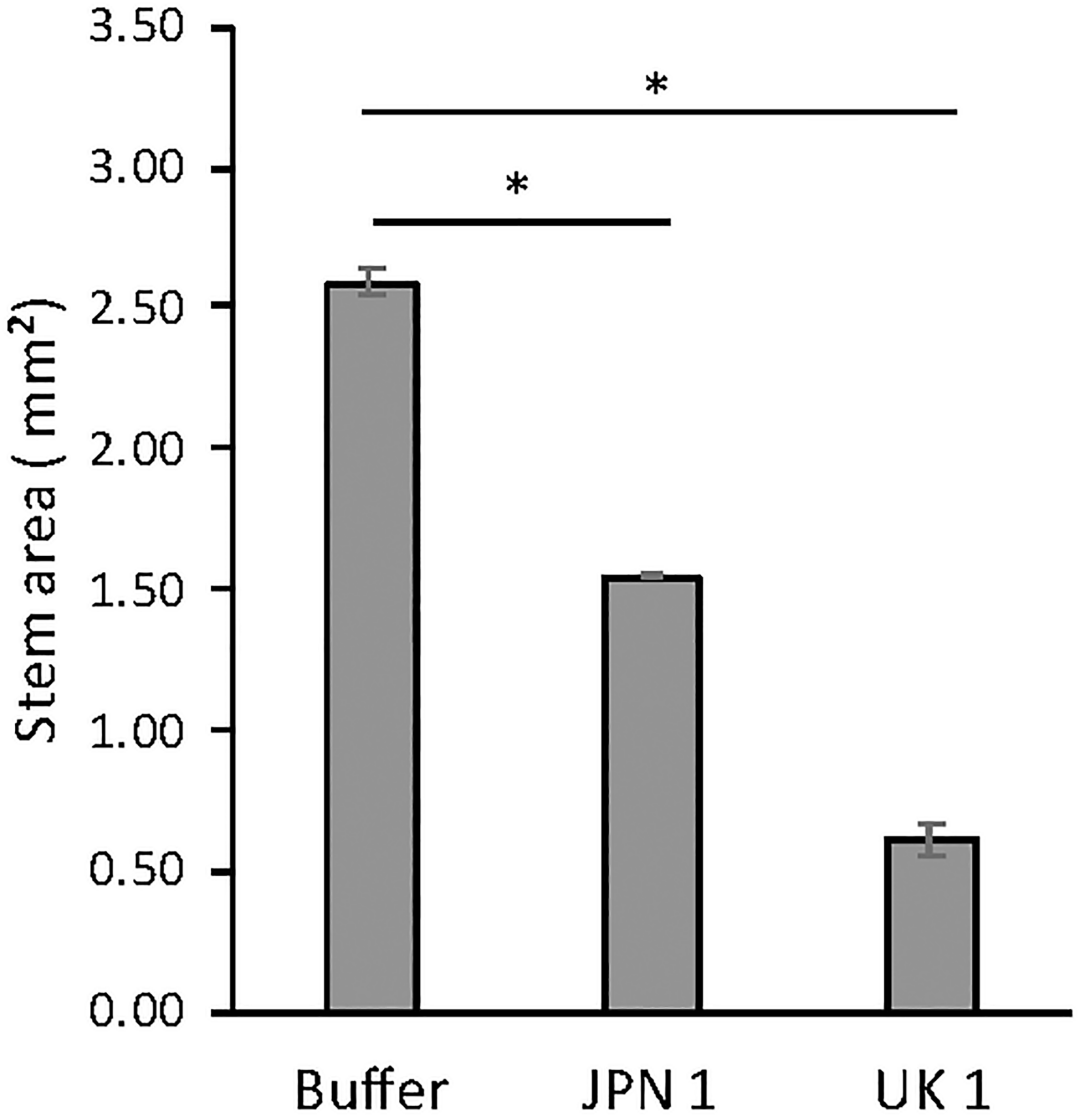
Inflorescence stem areas of non-infected and TuMV-infected Arabidopsis plants, showing statistically significant differences (marked with and asterisk) between infected and non-infected plants (see text for experimental details). The statistical analysis was a student’s *t*-test comparing Mock and JPN 1, and Mock and UK 1 (*p*<0.05).

The possible effect of TuMV infections on the inflorescence stem of Arabidopsis plants was assessed by comparing the cross-sectional areas of stems from the basal region (first internode) at 20days after infection (dai). In control (buffer-inoculated) plants, the sections were done 5cm above the rosette. UK 1-inoculated plants normally did not elongate 5cm, so these stems were sampled just below the first internode. The areas were obtained by image analysis of microscope examinations, using the software Fiji. Compared to control plants, both the JPN 1-and UK 1-inoculated had significantly reduced stem areas (Figure 1). The area was reduced from 2.59±0.04mm^2^ for the buffer-inoculated control stems to 1.54±0.01 for JPN 1 and 0.61±0.06 for UK 1 (mean+SEM). These results clearly showed that TuMV infections with either viral strain led to thinner inflorescence stems.

### Histochemical Analyses of the Virus-Infected Narrow Stems Reveal Viral Strain-Specific Alterations in Lignin SCW Deposition and in Stem Cell Sizes

**Figure 2:**
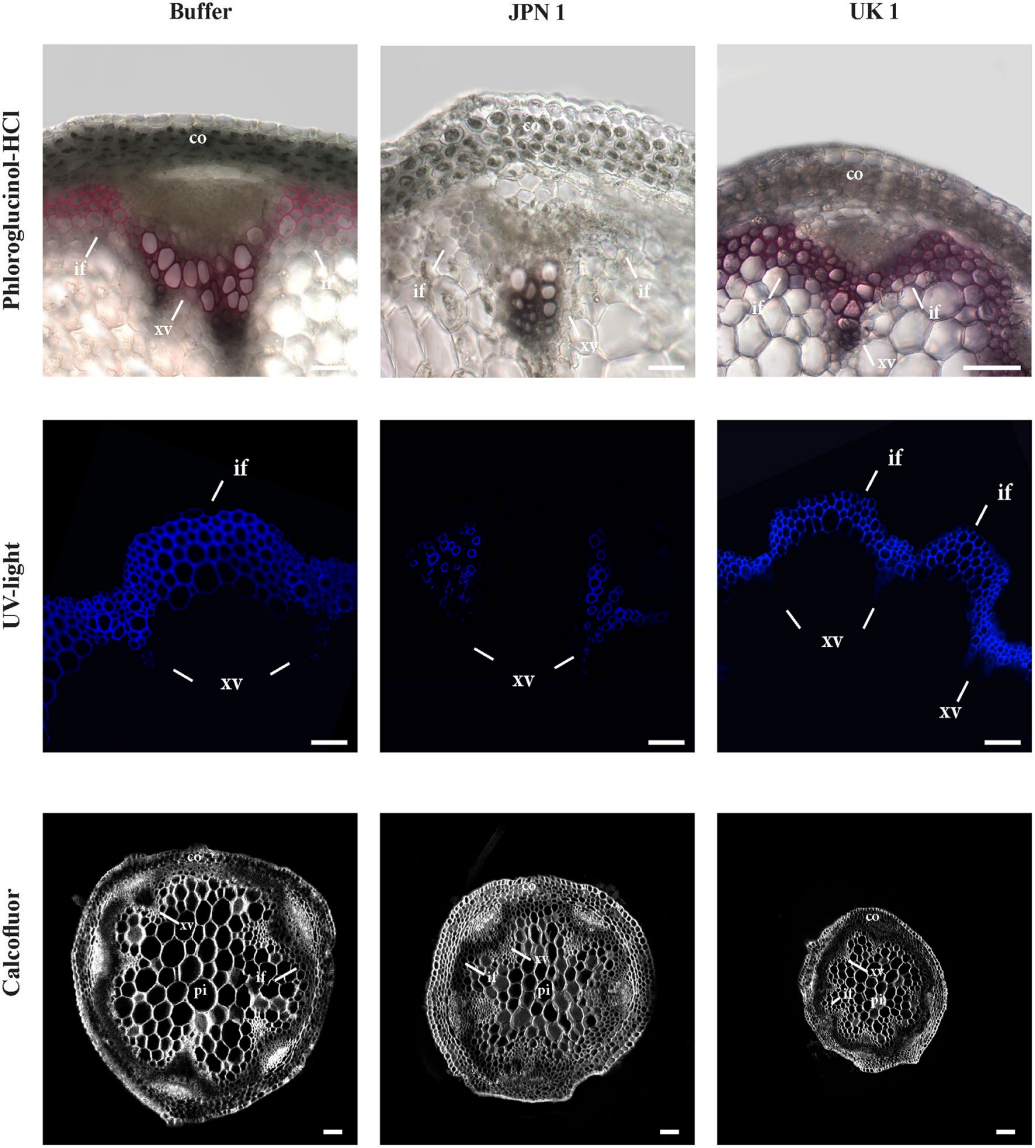
Lignin histochemical analyses of the secondary cell walls (SCWs) of the inflorescence stems from plants mock-inoculated (buffer) or inoculated with either TuMV strain (JPN 1 or UK (1). Top row shows lignin staining with phloroglucinol; middle row shows lignin intrinsic fluorescence upon ultraviolet excitation (UV light); and bottom row shows cellulose staining with Calcofluor. Bars indicate 100 μm. The cortex (co), xylem vessels (xv), and interfascicular fibers (if) are marked with letters. Note the lack of differentiation in the interfascicular fibers of JPN 1.

We reasoned that important alterations in the composition/deposition of the stem SCWs of virus-infected plants could be underlying their reduction in diameter, so we approached their characterization by means of histochemical analyses. The presence of lignin in the secondary walls was assessed by phloroglucinol-HCl staining (Figure 2, upper row). In contrast to buffer-inoculated controls and plants infected with the UK-1 strain, where lignin stained SCW of the vascular bundles and interfascicular fibers in purple red, lignin deposition was absent or very low in JPN 1-infected SCWs (Figure 2). Analogous results were obtained when the stems were stained with Toluidine Blue (Supplementary Figure S1). Lignin was also directly visualized under UV light (Figure 2, middle row), which revealed lignin was present in xylem vessels, but not in xylary fibers or interfascicular fibers in the JPN 1-infected stems. This is probably a major reason for their lack of rigidity and high stalk fragility. No such lignin alteration was found in UK 1-infected stalks, which had a lignin deposition similar to buffer-inoculated plants. A major difference, however, was found in the cell sizes in the UK 1-infected (note the different sizes of the 100μm bar in the Figure). In UK-1 plants, this is an important cause for the reduced stem areas of these plants.

Calcofluor staining was also performed to analyze possible alterations in the deposition of cellulose, callose, or other β-glucans. No major alterations for these wall components in virus-infected plants were found (Figure 2, bottom row), although the differences in cell sizes were also evident.

### Viral Strain-Specific Xylan Alterations Revealed by Immunofluorescence

**Figure 3:**
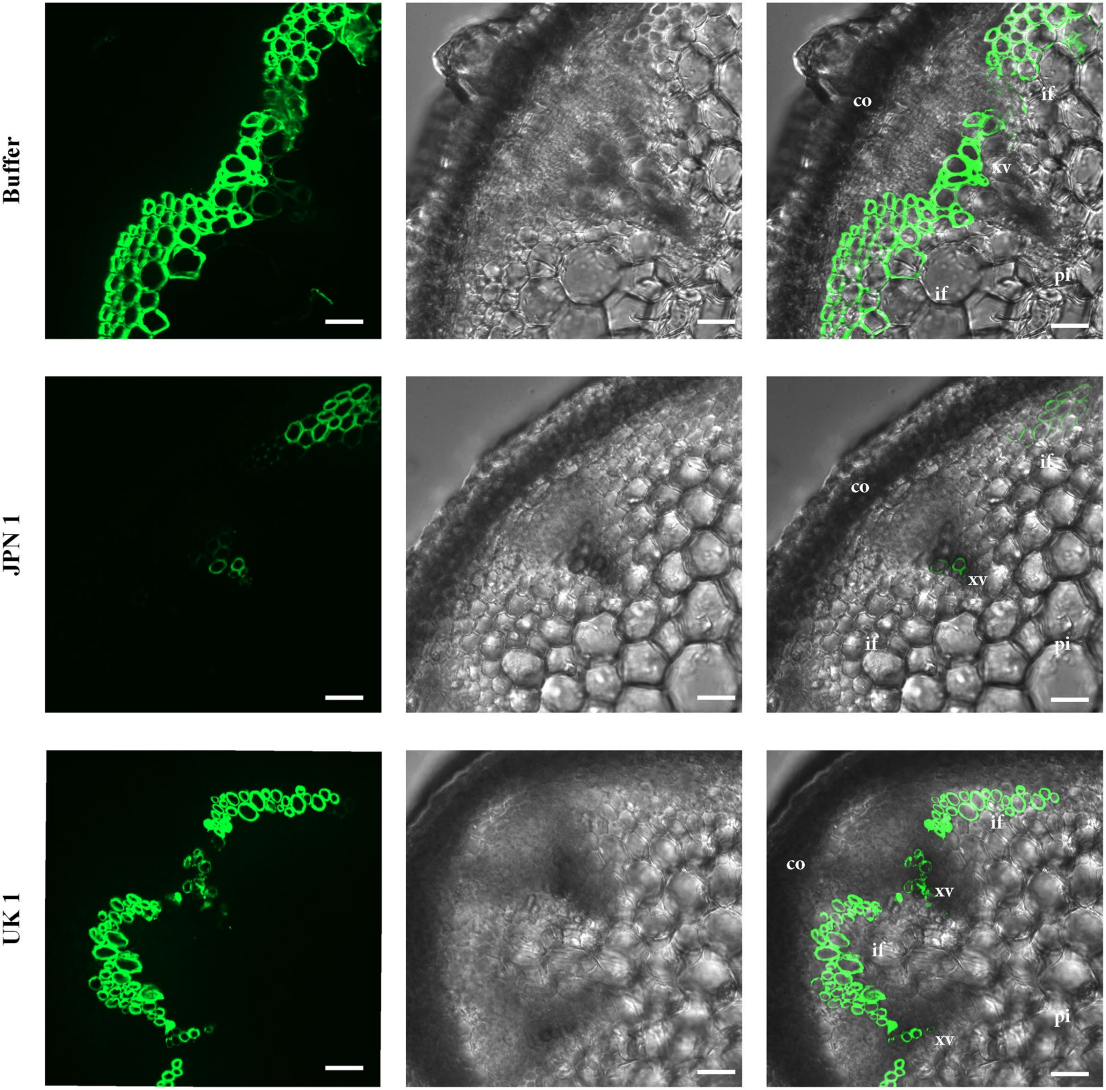
Xylan immunohistochemical analysis. The presence of xylan was revealed by its recognition by the monoclonal antibody LM10, followed by the reaction with a fluorescent secondary antibody. Xylan is only weakly detected in the xylem in TuMV strain JPN 1-infected plants (middle row) compared to buffer control (top) or strain UK 1 (bottom). In the third column, the fluorescence images are superimposed on the bright field shown in the second column. The cortex (co), xylem vessels (xv), interfascicular fibers (if), and pith (pi) are marked with letters.

A monoclonal antibody to xylan (LM10; (McCartney et al., 2005)) was used to analyze possible alterations in this important hemicellulose component of SCWs (Figure 3). The UV visualization of LM10 localization with a fluorescent secondary antibody showed a major lack of xylan in JPN 1-infected plants, with sparse fluorescent signal present only in the xylem vessels, and either no or irregular fluorescence in interfascicular fibers. No relevant xylan-label differences were found between the UK 1-plants and the buffer-inoculated, indicating a situation similar to the one found for lignin. The absence of xylan, and possibly of other hemicellulose components, would be contributing to the fragility and non-rigid inflorescence stems from JPN 1-infected plants, together with the lignin deficiency.

### The Viral Determinant for Inflorescence Stem Elongation Alone Does Not Determine the Creeping Habit of JPN 1-Infected Plants

The infection of Arabidopsis by the TuMV isolates leads to alterations in the inflorescence stem development. In the case of UK 1, the lack of stem elongation is the most dramatic one, whereas JPN 1 infections lead to fragile, non-erect stems conferring a creeping habit to the plant. The viral non-structural protein P3 was identified initially as the determinant for the lack of elongation in UK 1-infected Arabidopsis plants (Sánchez et al., 2015). Later, this determinant was finely mapped to the amino acid at position 279 of the protein (López-González et al., 2020). However, the determinant for the JPN 1-induced creeping phenotype has not been identified so far. Considering the differential intracellular ER-associated movement dynamics of the P3 proteins of the two isolates (López-González et al., 2020), we hypothesized that disrupted development of cells with thick cell walls could also be mediated by the P3 protein.

**Figure 4:**
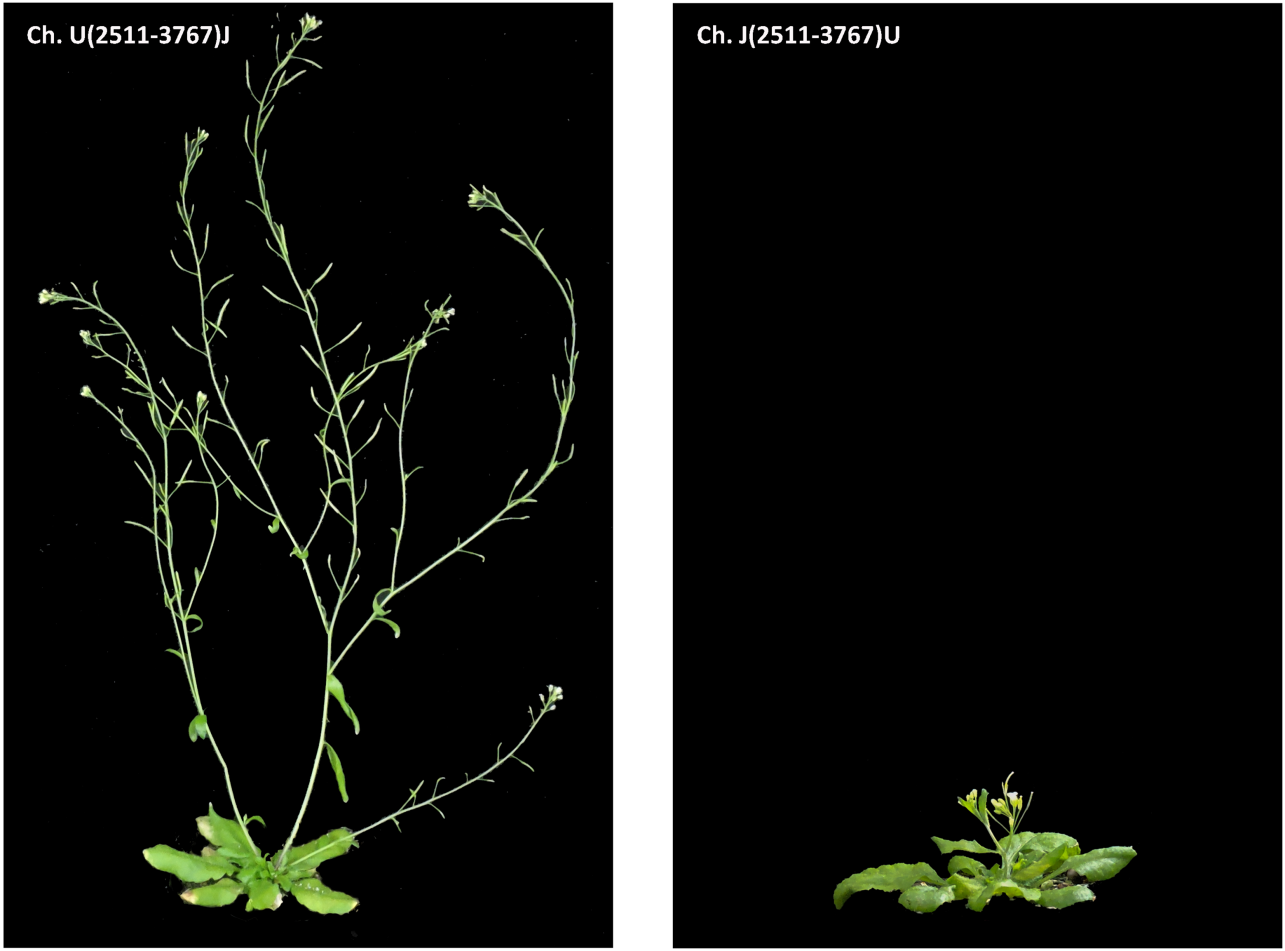
Phenotypes of Arabidopsis plants inoculated with viral chimeras (Sánchez et al., 2015) between TuMV isolates UK 1 and JPN 1, centered in the P3 region of the viral genome. The chimeras used for inoculation are shown in the panels.

To tackle this issue, we used the same viral chimera approach described previously (Sánchez et al., 2015), where the P3 cistron was interchanged between the two isolates. The viral chimeras were Ch. U(2511-3767)J, where the JPN 1 P3 was inserted into the UK 1 strain and Ch. J(2511-3767)U, which was the reverse with UK 1 as the donor inserted into JPN 1. These chimeras were inoculated to Arabidopsis plants and the creeping habit trait was examined. As predicted, interchanging the P3 viral proteins changed the elongation trait, and plants infected by Ch. J(2511-3767)U, which is mostly JPN 1 with a UK 1 P3, did not elongate a stem, whereas the reciprocal chimera Ch. U(2511-3767)J did. Surprisingly, in opposition to its parent isolate JPN 1, plants infected with the Ch. U(2511-3767)J chimera did not show a creeping habit and produced an erect stalk (Figure 4). This discarded the P3 protein as the sole viral determinant, although did not discard the possibility of its implication in the trait in a joint action with other factor(s), as yet unidentified.

**Figure 5:**
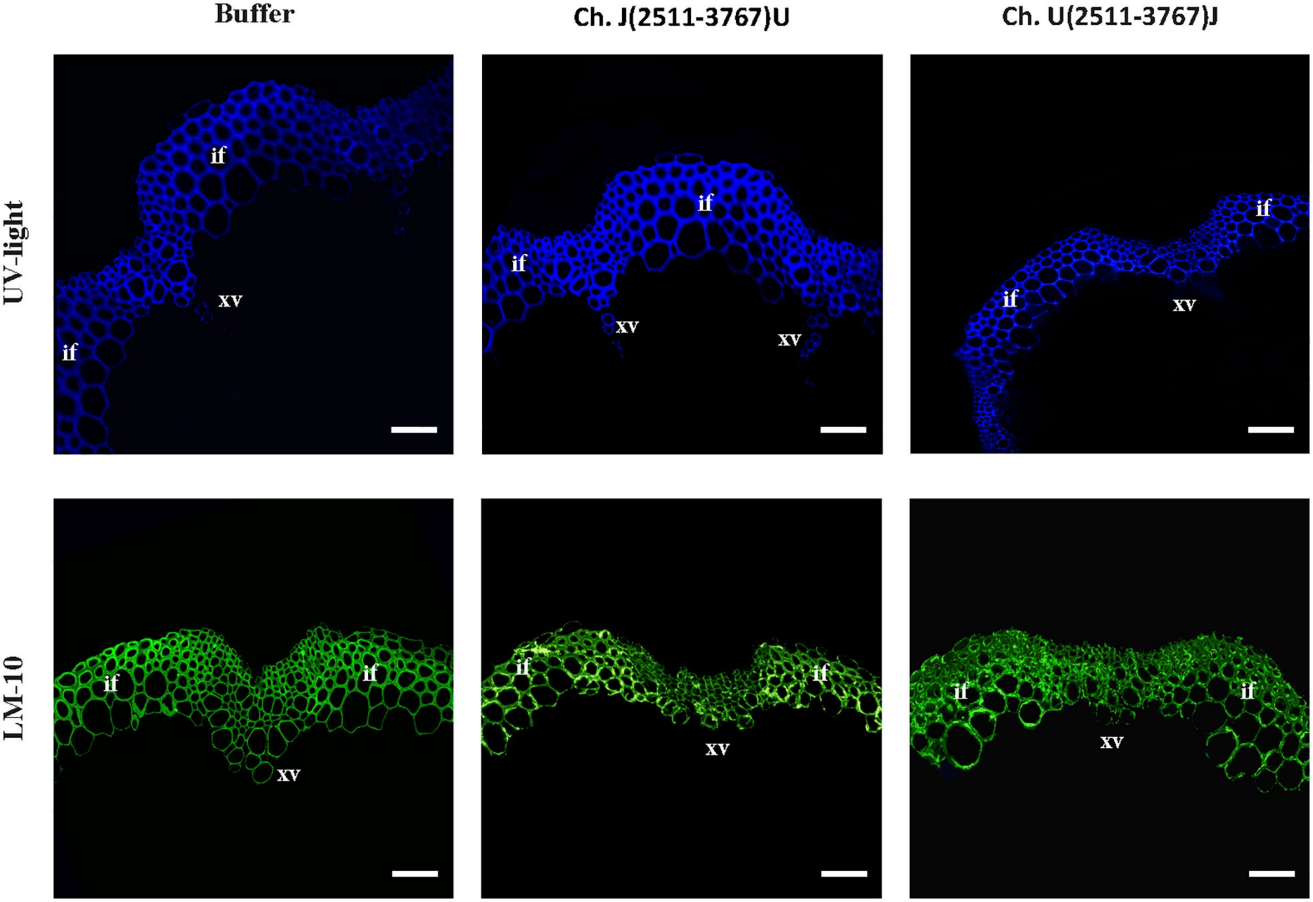
Visualization of lignin by UV (upper row) and xylan (monoclonal antibody LM 10, bottom row) in the inflorescence stem SCW of buffer-inoculated or TuMV chimeras centered in the P3 region of the viral genome. Bars indicate 100 μm. Xylem vessels (xv) and interfascicular fibers (if) are marked.

The visual observation of the phenotypes of plants infected with the viral chimeras was complemented with a histochemical analysis to assess possible alterations in the stem development and SCWs. The results shown in Figure 5 revealed that lignin and xylan-label of SCWs were similar among the controls and chimeras. These results indicate that JPN 1 P3 by itself is not enough determinant within the viral genome to induce the SCWs alterations but is required for them because the alterations disappear with UK 1 P3.

### Gene Expression Analysis of the Master Genes Regulating SCW Biosynthesis in TuMV-Infected Arabidopsis Plants

The biosynthesis of the SCW is a highly complex process regulated at several levels (McCahill and Hazen, 2019). Among these levels, the transcriptional one is central and has been the subject of intense research over the last two decades or so (Yamaguchi et al., 2011; Schuetz et al., 2013; Ohtani and Demura, 2019). A global view of the regulation at the transcriptional level reveals a network of feed-forward loops affecting both regulatory genes (transcription factors, TFs) and metabolic genes (Taylor-Teeples et al., 2015). Occupying a central position in the network is TFs MYB46 and MYB83. These are involved in the regulation of the TFs that regulate the expression of the genes required to biosynthesize the structural components of SCWs. Upstream of MYB46 and MYB83 are the master switches for determining fiber, protoxylem, and metaxylem differentiation. The master regulators for fiber cell fate differentiation are SND1/NST3 and NST1 (Zhong et al., 2006; Mitsuda et al., 2007). For xylem vessels in Arabidopsis, VND7 triggers protoxylem and VND6 triggers metaxylem tracheary cell fate, respectively (Kubo et al., 2005; Yamaguchi et al., 2011). We therefore decided to evaluate the expression of these transcription factors. Moreover, due to the observed lack of xylan accumulation in JPN 1 inflorescence stems, we also evaluated the expression of the *IRX9* and *IRX10* genes which encode glycosyltransferases involved in the synthesis of the xylan backbone. *INTERFASCICULAR FIBERLESS* (*IFL1)* was also analyzed because Arabidopsis mutant *ifl1* closely mimics the loss of fibers phenotype seen in JPN 1 (Zhong and Ye, 1999). This gene, also called *REVOLUTA* (Ratcliffe et al., 2000), encodes a TF of the class III HD-ZIP family with complex roles in plant development (Ramachandran et al., 2017).

**Figure 6:**
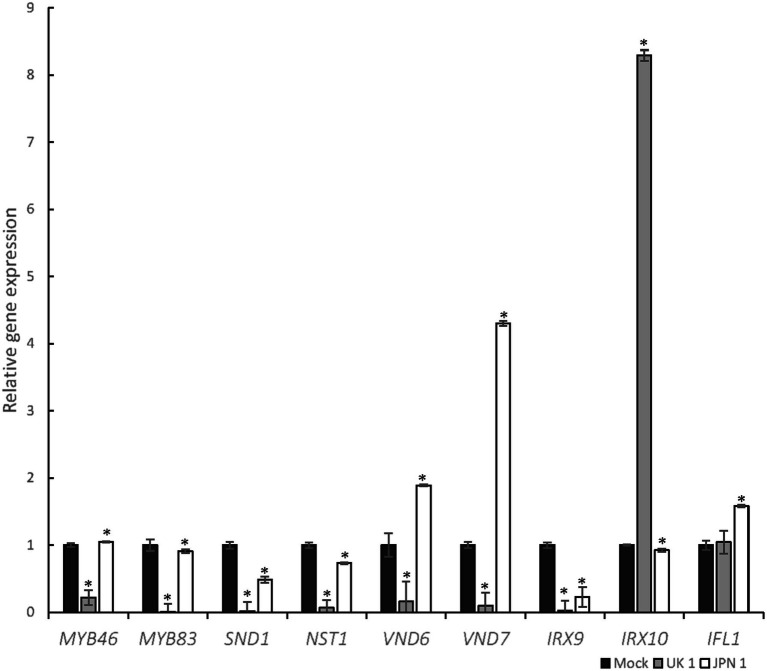
Transcriptomic analysis of selected genes involved in SCW biosynthesis after TuMV infection. The graph shows the results of RT-qPCR of selected genes, represented as relative gene expression in JPN-1-infected or UK-1-infected strains of TuMV, compared to mock-inoculated plants. Comparisons are pairwise of each virus-infected and mock-inoculated. Genes analyzed and color-coded bars are shown in the figure. Asterisk indicates a significant difference compared with wild type (^*^p<0.05 by *t*-test). Error bars show standard deviation.

The results obtained after RT-qPCR of the genes encoding the TFs and enzymes mentioned above are shown in Figure 6. A first global view of the virus-induced transcriptomic alterations notes that UK 1had a higher quantitative impact on the expression of almost all genes under analysis, which is not surprising given the impaired elongation of the stem in these plants. This included both downregulation and a surprising upregulation in *IRX10*. In plants infected with JPN 1, where the fiber development was impaired (Figures 2, 3), the fiber master regulator *SND1/NST3* was the most strongly downregulated gene. The two xylan biosynthetic genes analyzed (*IRX9* and *IRX10*) were also decreased in these lines, consistent with the loss of LM10 staining seen in Figure 3. The vascular-related NAC domain TF genes (*VND6* and *VND7*) had the opposite behavior, increasing with JPN 1 and decreasing with UK 1. This may reflect the relatively normal xylem vessels seen in JPN 1, in contrast to the fiber phenotype, and the decreased vasculature in the poorly elongated UK 1. The results obtained are consistent with the phenotypic and microscopic observations of TuMV-infected Arabidopsis plants, since they reveal the high impact that TuMV infections have on the expression of the main genes involved in the regulation of the SCW formation.

### The Impact of TuMV Infections on Plant Fertility Is Also Viral Strain Specific and Can Be Related to Reduced Endothecium Lignification for JPN 1-Infected Flowers

**Figure 7:**
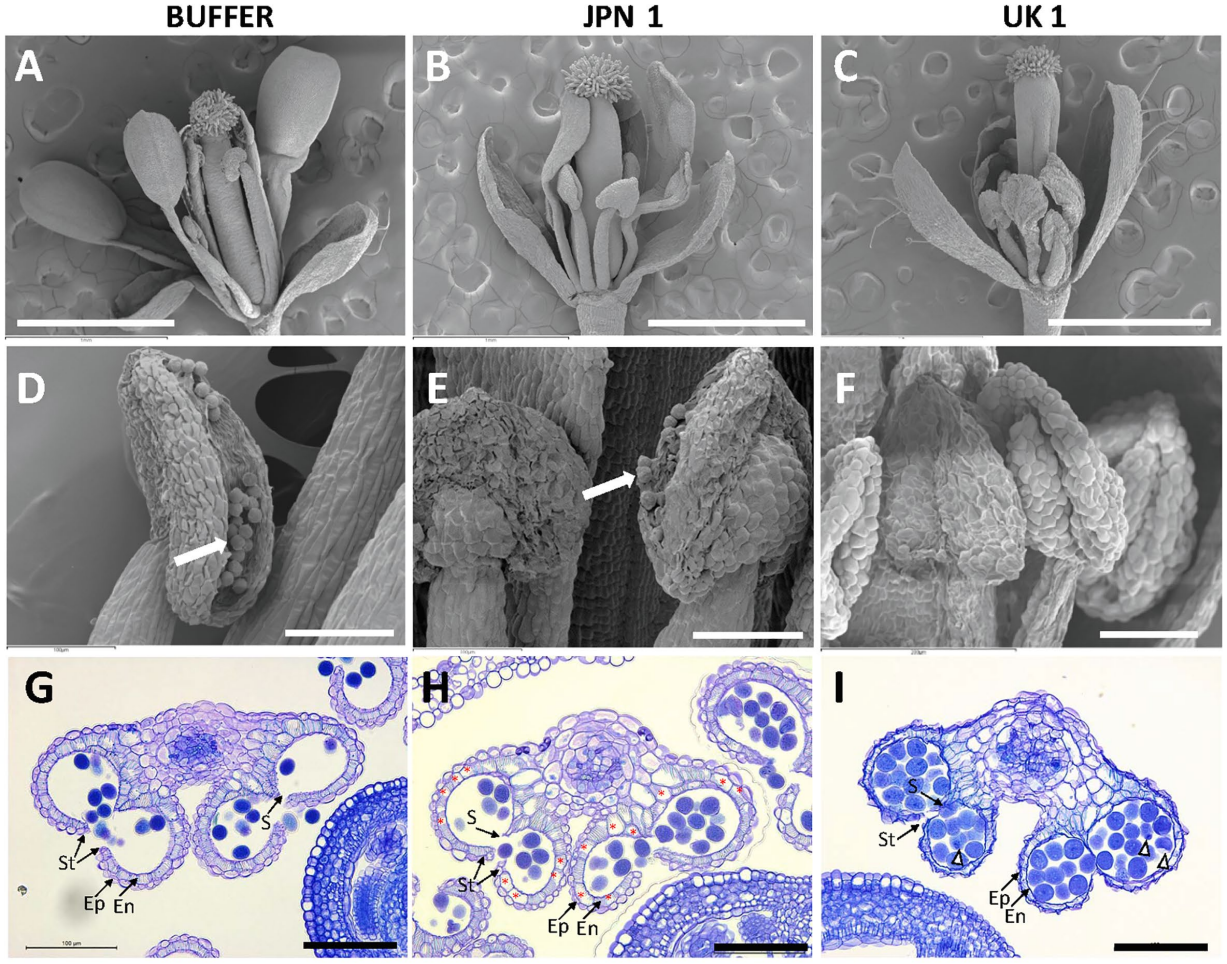
Phenotypes of flowers from control buffer-inoculated and two strains of TuMV-infected Arabidopsis plants. Scanning electron micrographs of flowers at anthesis **(A-C)** and flower anthers **(D-F)**. Pollen grains are visible in D and E (arrows). Transversal sections of anthers from flowers at anthesis stained with toluidine blue **(G-I)**. Non-infected mock plants show strong lignification of the endothecium. Endothecium cells showing absence of lignification are marked by asterisks in H. Abnormal pollen grains are marked by arrow heads. Scale bars indicate: 1mm in A-C and 100μm in D-I. Epidermis (Ep); endothecium (En); stomium (St); and septum (S).

Arabidopsis reproductive development is largely affected by TuMV infections with both JPN 1 and UK 1 isolates, although the alterations are usually more striking in UK 1-infected plants (Sánchez et al., 2015). JPN 1-infected plants produce a reduced amount of pollen, whereas UK 1-infected plants mostly remain non-fertile. The light microscopy observations sustaining these effects have now been deepened to further detail by scanning electron microscopy (SEM) examinations (Figure 7). In comparison with buffer-inoculated plants (Figures 7A,D), flowers from JPN 1-infected plants showed shorter stamens and lower amount of pollen (Figures 7B,E). Petal and stamen development did not elongate in UK 1-inoculated plants showing signs of premature senescence (Figures 7C,F). In these plants, anthers remain immature and pollen is not usually seen (Figure 7F).

Following development of male gametophytes (pollen grains) within the anther, successful plant reproduction requires the dehiscence of the anther to release the mature pollen. Anther dehiscence entails the development of lignified secondary walls in endothecial cells (Goldberg et al., 1993). We assessed endothecium lignification of anthers by means of histological section of flowers at anthesis. The flowers from buffer-inoculated plants showed a continuous layer of lignified cells in the endothecium below the epidermis, degradation of septum cells, and release of pollen grains (Figure 7G). Anthers from JPN 1-inoculated plants showed reduced endothecium lignification, although some dehiscence occurred and the anthers were able to release the pollen to some extent (Figure 7H). In contrast, the anther from UK 1-inoculated plants rarely dehisced, the epidermis and endothecium layers were not clearly differentiated and showed signs of degeneration. Moreover, pollen grains showed internal defects and signs of degradation (Figure 7I).

Thus, JPN 1 isolate has a specific effect on SCW lignification also during reproductive development, whereas UK 1 has a general effect on flower development that compromises plant fertility.

## Discussion

A role for virus-induced modifications of plant cell wall structure has long been recognized. Plant viruses encode proteins (movement proteins, MPs) whose main function is to promote and mediate virus intercellular movement, thus propagating the infection to still uninfected cells (Dorokhov et al., 2020; Kumar and Dasgupta, 2021). In order to move, viruses need to overcome the formidable barrier of the wall and mostly do it by increasing the size exclusion limit (SEL) of wall-crossing plasmodesmata. This increase implies modification of the cell wall, mostly through MP-induced degradation of the callose regulating the SEL of plasmodesmata. In addition to this well-known phenomenon, recent work has proposed cell wall modifications by regulation of cell wall structural proteins. However, these modifications, very recently reviewed (Kozieł et al., 2021), affect the primary cell wall and do not have a direct impact on specific plant developmental traits. The work described in this paper deals with effects of virus infections on the SCW, a topic unexplored so far, and their reflection on plant development.

The effects of TuMV infections on Arabidopsis developmental traits, previously described (Sánchez et al., 2015), are most dramatic on the elongation, erection, fragility, and branching of the inflorescence stem, and on flower fertility. A simple quantitative analysis of the inflorescence stem areas showed that they were significantly narrower in infected plants, suggesting some virus effect on the correct formation of the thick SCW deposited on specialized cells of the stem. One of the TuMV strains (JPN 1) induced significantly less area reduction than the other (UK 1), indicating likely differential effects. The presumed differences were confirmed upon histochemical analyses of stem cuts. Different staining approaches uncovered that lignin deposition was deeply altered in xylary fibers or interfascicular fibers of JPN 1-infected plants. Similar alterations were found for xylan deposition. On the contrary, and somewhat unexpectedly, none of these alterations were found in UK 1-infected plants, which bore the narrowest stems. However, stained UK 1 cells appeared as abnormally small in the analyses, which indicated severe impairment in cell development, and fully justified the strong stem area reduction. Our first microscopic observations of the effects of TuMV on stem development (Sánchez et al., 2015) had already detected a cellular basis of the alterations, including size. Those analyses and the present ones are not directly comparable because they were done on early developing stems, whereas now fully developed ones (first internode above the rosette) were analyzed. However, important cell alterations could already be found then, now confirmed and expanded, which are on the basis of the macroscopic developmental defects in stem elongation and erection. The SCW defects imposed by JPN 1 would justify the lack of upright growth and fragility in stems that, nevertheless, are able to elongate. A more general cell developmental defect seems to be causing the lack of stem elongation in UK-1 infected plants.

The defects in flower development and fertility had also been already noticed in our previous work. Like for the inflorescence stem, the new microscopic analyses shed light on the cellular basis of the alterations. Very few flowers (if any) form in the practically stemless UK 1-infected plants. Their petals and stamens barely elongate, and their anthers do not release visible pollen, or do it in minute amounts. The SEM analyses provided further details and revealed a general alteration in the development of the different flower organs and pollen maturity in UK 1-infected plants. In the case of the JPN 1-infected, important defects in the SCW formation could be identified, especially in endothecium cells, which showed a low lignification degree. There seems to be a good correlation between the defects in SCW and the decrease in pollen release in the JPN 1-infected, whereas the almost absolute absence of pollen in UK 1-infected plants appears to be more related to pollen grain degradation and lack of clear differentiation of endothecial cells, again compatible with more general plant developmental defects.

A previous transcriptomic analysis performed on total RNA of the aerial part of UK 1-infected plants (Sánchez et al., 2015) revealed massive changes in plants which had already reached stage 5.1 of development (Boyes et al., 2001), affecting the transcription level of over 1,000 genes, a result fully compatible with an interpretation of alterations affecting multiple levels and pathways in plant development. The situation is quite different in JPN 1-infected plants, which showed transcriptional alterations in just over 60 genes at a similar developmental stage, indicating that developmental alterations induced by JPN 1 are less massive, probably affecting mostly just specific development aspects. As shown by our new analyses, one of these aspects is SCW deposition, both in the inflorescence stem and the endothecial cells of the flowers.

The new transcriptomic analysis focused on genes involved in stem SCW formation in the first internode of TuMV-infected plants. Significant changes were found in the genes analyzed, yet with different patterns for the two viral strains. In the case of UK 1, the expression of most regulatory genes (transcription factors, TFs) dropped down drastically, with the only exception of *IFL1*, which is not a TF only and specifically involved in the control of SCW formation. Rather, its role is more related to general patterning control, mostly through the control of auxin biosynthesis and fluxes (Brandt et al., 2014). In UK 1-infected plants, even though no obvious histological defects were found in SCW, the strong decrease found in specific SCW-controlling genes indicates that the process of SCW deposition is also part of the global developmental alteration. Maybe some other aspect of its regulation could be playing a role. Remarkable opposite alterations were found in the expression of the two biosynthetic genes analyzed (*IRX9* and *IRX10*). Most likely, a compensatory effect underlies this result. In JPN 1-infected plants, a completely different view was obtained. Here, the expression of the two TFs involved in fiber cell fate differentiation is the most decreased, in line with the fiber SCW impairment in these plants. No major differences were found for the two central *MYB* controlling genes, suggesting that the most relevant origins of the alterations found lie somewhere else, at the level of cell-type determination. This view is reinforced by the increase in the expression of the VND TFs (*VND6* and *VND7*), involved in protoxylem and metaxylem determination, considering that no important defects were found in the xylem vasculature in JPN 1-infected plants. Transcription of the biosynthetic genes is decreased, especially *IRX9*, in line with the SCW alterations found. *IFL1* transcription increased, but the interpretation of this result is less obvious given its more general role.

In an effort to link the SCW defects found in this work, and the possibly related creeping habit trait of JPN 1-infected plants, a study involving viral chimeras was undertaken. The approach taken was centered on the viral P3 protein, given its determinant role for the lack of stem elongation in UK 1-infected plants (Sánchez et al., 2015; López-González et al., 2020). The results obtained allowed further dissection of the TuMV-induced developmental alterations. Interchanging the P3 coding regions of both viral strains in their respective infectious clones confirmed again the involvement of this viral protein in stem elongation. However, the characteristic creeping habit of the JPN 1-infected was not found in plants infected with chimera Ch. U(2511-3767)J, in which the P3 of JPN 1 replaced the UK 1 P3. Instead, plants with normal erect inflorescence stems were obtained. The histological inspection of plants infected with this viral chimera, or with its reverse Ch. J(2511-3767)U, revealed no alterations in SCW composition induced by any of the chimeras. So, the results indicate that UK 1 P3 in a TuMV genome is enough to arrest elongation (in fact a single a.a. in the protein is the determinant), although it does not affect SCW composition, but a simple replacement of UK 1 P3 with JPN 1 P3 is not enough to induce a creeping habit, nor to affect SCW composition. Thus, defects in SCW composition and a creeping habit appear as two linked characteristics, as expected, but elongation and upright growth can happen together in TuMV-infected plants. They must then be seen as two different and unlinked characteristics of JPN 1-infected plants. It follows that if the P3 protein is a determinant of a creeping habit, it is only a partial one, requiring the concerted action of another, still unidentified, extra viral determinant.

How the effects of the two TuMV strains on plant and cell development and SCW formation relate to the subcellular localization and behavior of their P3 proteins, is still a question to be answered. TuMV P3 is a peripheral ER-membrane protein which undergoes a process of ER-streaming toward the cell periphery, a process related to its role as a protein involved in viral intercellular movement (López-González et al., 2020). This is common to the P3 proteins of both viral strains, but they also exhibit important differences. One is the much faster rate of ER-streaming in UK 1. A single amino acid at position 279 is the responsible for both streaming rate and the arrest of stem elongation, strongly suggesting that these are two associated processes. Another important difference is the co-localization of P3 with another viral protein, 6K2. In addition to its ER localization, UK 1 P3 can be found co-localizing together with 6K2 in ER-derived 6K2-induced vesicles and chloroplasts in the nuclear periphery. This association is not found for JPN 1 P3. As mentioned above, the typical UK 1 stemless and sterile flower phenotypes go together with the UK 1 P3 subcellular behavior, but the new results show that SCW composition or final cell deposition is not altered in these plants. Rather, important defects in cell size and/or maturation are found. It is not obvious to envision how the typical subcellular behavior of UK 1 P3 can have such an important effect on plant developmental traits, at both macroscopic and cellular levels. ER-streaming is common to both P3 proteins, and their difference in speed does not seem “*a priori*” a factor so important to justify them. It is possible that its association with the 6K2-induced vesicles promotes much more important rearrangements in the intracellular endomembrane system than in the case of JPN 1 P3, and this could be at the basis of all subsequent defects. It is a point requiring further investigation. As to JPN 1 P3, we had previously suggested that its subcellular behavior could be linked to the creeping habit of the infected plants, but the new results with the viral chimeras show that this is not the case. The defects in SCW are found but, as mentioned above, these are not only influenced by the presence of a JPN 1 P3. Again, further work is needed to identify additional viral factors.

A final consideration concerning the different behaviors of TuMV strains is worth remarking. UK 1 and JPN 1 are considered as representative of two strains, not so closely genetically related, originally named MB and MR and also renamed as World B and Asian BR, respectively (Ohshima et al., 2002; Sánchez et al., 2003; Tomimura et al., 2003). In the recent years, several pieces of evidence have been found supporting the view that different TuMV strains (and maybe even different viral isolates within a strain) establish quite different relationships with the infected host. Thus, not only the different subcellular behavior of the P3 protein, but also the different impact of the infections on plant development and/or SCW formation discussed in this paper happen. Non-host resistance range also differs between strains (Sardaru et al., 2018), and recent important differences have been recently described for the interaction of the P1 viral protein with the host protein G3BP-2, leading to relevant differences in the formation of stress granules (Reuper and Krenz, 2021). How these relevant differences in the biology of intimate interactions with the host should be taken into account for the taxonomical differentiation between strains of viral species, will need to be considered in the future. Perhaps, these biological differences should be enough to consider them as separated viral species.

## Data Availability Statement

The original contributions presented in the study are included in the article/Supplementary Material, further inquiries can be directed to the corresponding author.

## Author Contributions

SL-G and CG-M run the experiments. FS, MS, and AS supervised different parts of the experimental work. FP conceived the work with the help of the other authors, supervised the whole work, and wrote the script with contributions from the other authors. All authors read and approved the final form of the script.

## Funding

The work at the CBGP was funded by several INIA grants. During the course of the work SL-G was funded by a predoctoral FPI-INIA fellowship/contract. We thank the Spanish Ministry of Science for the Severo Ochoa Excellence Accreditations to the CBGP (SEV-2016-0672).

## Conflict of Interest

The authors declare that the research was conducted in the absence of any commercial or financial relationships that could be construed as a potential conflict of interest.

## Publisher’s Note

All claims expressed in this article are solely those of the authors and do not necessarily represent those of their affiliated organizations, or those of the publisher, the editors and the reviewers. Any product that may be evaluated in this article, or claim that may be made by its manufacturer, is not guaranteed or endorsed by the publisher.

## Supplementary Material

**Supplementary Figure S1 |** Toluidine-blue lignin histochemical analyses of the secondary cell walls of the inflorescence stems. The virus or buffer inoculum are indicated. Bars indicate 100μm. The cortex(co), xylem vessels (xv), and interfascicular fibres (if) are marked with letters. The results with Toluidine-blue reinforce those shown in Figure 2.
