# Supplementary figures and images for: The Effects of Turnip Mosaic Virus Infections on the Deposition of Secondary Cell Walls and Developmental Defects in Arabidopsis Plants Are Virus-Strain Specific
Source: Front Plant Sci. 2021 Oct 8;12:741050. doi: 10.3389/fpls.2021.741050 (PMC8531753; doi:10.3389/fpls.2021.741050)

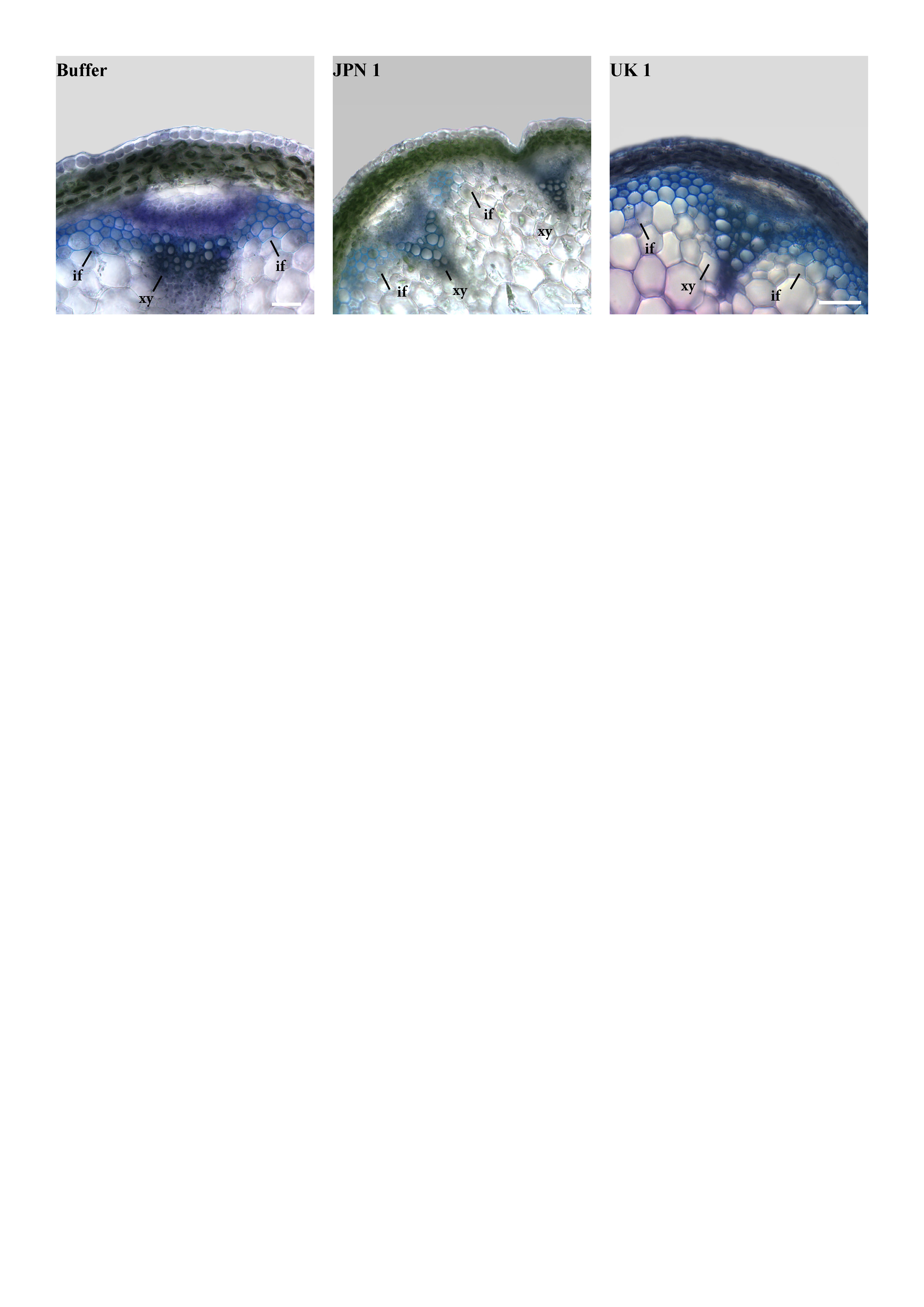

Supplement: Supplementary file 1 [file image_1.TIFF]
