# Supplementary material for: The Effects of Turnip Mosaic Virus Infections on the Deposition of Secondary Cell Walls and Developmental Defects in Arabidopsis Plants Are Virus-Strain Specific
Source: Front Plant Sci. 2021 Oct 8;12:741050. doi: 10.3389/fpls.2021.741050 (PMC8531753; doi:10.3389/fpls.2021.741050)
Supplement: Supplementary file 2 [file Table_1.DOCX]

**Table S1. Primers used for RT-qPCR**

| Primer | Gene | Sequence (5’-3’) |
| --- | --- | --- |
| IFL 1 (F)  IFL 1 (R) | AT5G60690 | CTTTGCTTATCCGGGAATGA  CCCGGTACAAATCTGAAGGA |
| IRX 9 (F)  IRX 9 (R) | AT2G37090 | GCGAGAAGCTGAAGGAGAAA  CCGAGTGTTTTTCCACGACT |
| IRX 10 (F)  IRX 10 (R) | AT1G27440 | AATCGGACTGAAGGAGCTGA  GCGTGGAATATCTGGAGGAA |
| MYB46 (F)  MYB46 (R) | AT5G12870 | GGCAACAGGTGGTCTCAGA  AGAGGAATCGCTTGCTGTGT |
| MYB83 (F)  MYB83 (R) | AT3G08500 | AGAGACAAAGGCAAGCCAAA  AGCGAAGGCGACAACTTTTA |
| NST 1 (F)  NST 1 (R) | AT2G46770 | TTACTAGCTGGGCGGCTTTA  CATAGCTCCATCTCCGGTGT |
| SND 1 (F)  SND 1 (R) | AT1G32770 | GCTACCGGACGTGACAAAAT  CCACCCAACCTTCTTCGTTA |
| VND 6 (F)  VND 6 (R) | AT5G62380 | CTAACTGGTTCACGCAGCAA  TGACCATCATCTCGTTGCAT |
| VND 7 (F)  VND 7 (R) | AT1G71930 | GAAGCCAATTCCAAACCAGA  TGATGCATCGATGAGGATGT |
| ACTIN 8 (F)  ACTIN 8 (R) | AT1G49240 | AACCCAAAAGCCAACAGAGA  TGGAAGTGAGAAACCCTCGT |
